# Sepsis is associated with mitochondrial DNA damage and a reduced mitochondrial mass in the kidney of patients with sepsis-AKI

**DOI:** 10.1186/s13054-020-03424-1

**Published:** 2021-01-25

**Authors:** Elisabeth C. van der Slikke, Bastiaan S. Star, Matijs van Meurs, Robert H. Henning, Jill Moser, Hjalmar R. Bouma

**Affiliations:** 1 Department of Clinical Pharmacy and Pharmacology, University of Groningen, University Medical Center Groningen, P.O. Box 30.001, EB70, 9700 RB Groningen, The Netherlands; 2 Department of Critical Care, University of Groningen, University Medical Center Groningen, Groningen, The Netherlands; 3 Department of Pathology and Medical Biology, Medical Biology Section, University of Groningen, University Medical Center Groningen, Groningen, The Netherlands; 4 Department of Internal Medicine, University of Groningen, University Medical Center Groningen, Groningen, The Netherlands

**Keywords:** Sepsis, Acute kidney injury, Reactive oxygen species, Mitochondria

## Abstract

**Background:**

Sepsis is a life-threatening condition accompanied by organ dysfunction subsequent to a dysregulated host response to infection. Up to 60% of patients with sepsis develop acute kidney injury (AKI), which is associated with a poor clinical outcome. The pathophysiology of sepsis-associated AKI (sepsis-AKI) remains incompletely understood, but mitochondria have emerged as key players in the pathogenesis. Therefore, our aim was to identify mitochondrial damage in patients with sepsis-AKI.

**Methods:**

We conducted a clinical laboratory study using “warm” postmortem biopsies from sepsis-associated AKI patients from a university teaching hospital. Biopsies were taken from adult patients (*n* = 14) who died of sepsis with AKI at the intensive care unit (ICU) and control patients (*n* = 12) undergoing tumor nephrectomy. To define the mechanisms of the mitochondrial contribution to the pathogenesis of sepsis-AKI, we explored mRNA and DNA expression of mitochondrial quality mechanism pathways, DNA oxidation and mitochondrial DNA (mtDNA) integrity in renal biopsies from sepsis-AKI patients and control subjects. Next, we induced human umbilical vein endothelial cells (HUVECs) with lipopolysaccharide (LPS) for 48 h to mimic sepsis and validate our results in vitro.

**Results:**

Compared to control subjects, sepsis-AKI patients had upregulated mRNA expression of oxidative damage markers, excess mitochondrial DNA damage and lower mitochondrial mass. Sepsis-AKI patients had lower mRNA expression of mitochondrial quality markers *TFAM*, *PINK1* and *PARKIN*, but not of *MFN2* and *DRP1*. Oxidative DNA damage was present in the cytosol of tubular epithelial cells in the kidney of sepsis-AKI patients, whereas it was almost absent in biopsies from control subjects. Oxidative DNA damage co-localized with both the nuclei and mitochondria. Accordingly, HUVECs induced with LPS for 48 h showed an increased *mnSOD* expression, a decreased *TFAM* expression and higher mtDNA damage levels.

**Conclusion:**

Sepsis-AKI induces mitochondrial DNA damage in the human kidney, without upregulation of mitochondrial quality control mechanisms, which likely resulted in a reduction in mitochondrial mass.
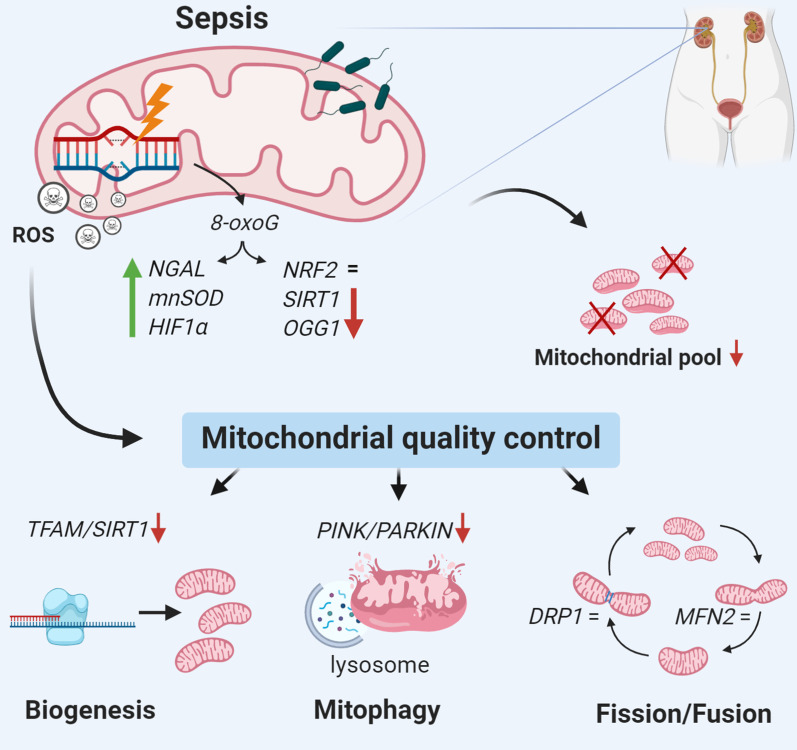

## Introduction

Sepsis is defined as a dysregulated host response to infection, which can lead to loss of organ homeostasis, multiple organ failure, and ultimately, death in patients [1]. Mortality and morbidity associated with sepsis and septic shock are high. In-hospital mortality rate amounts to 20–30% for sepsis and 40–60% for septic shock [1, 2]. Despite extensive research, the pathophysiology of sepsis remains incompletely understood. As a result, therapeutic options directed at the molecular cause of organ dysfunction are lacking, and the current therapy is limited to source control (i.e., antibiotics, drainage) and organ support [1–4]. Up to 60% of patients with sepsis develop acute kidney injury (AKI) and sepsis is the most common cause of AKI in the intensive care unit (ICU) [5, 6]. The occurrence of AKI in sepsis is associated with failure of other organs and increased mortality [5, 6]. Thus, sepsis-AKI constitutes a serious medical health problem.

Mitochondria have emerged as key players in the pathogenesis of sepsis-AKI [7–9]. Healthy mitochondria are essential for maintaining renal homeostasis and are important in supporting the metabolic challenge during sepsis [2, 7, 8]. Mitochondrial failure during sepsis results in renal ATP-depletion and increased levels of reactive oxygen species (ROS), which progresses into loss of cellular homeostasis and organ dysfunction [10]. Sepsis lowers ATP levels, increases biomarkers for mitochondrial dysfunction and reduces antioxidant defense, which are associated with poor survival, as demonstrated in muscle biopsies in 16 critically ill patients compared to 10 healthy, age-matched patients undergoing elective hip surgery [11]. It is known that accumulating levels of ROS during sepsis can cause damage to mitochondrial proteins and DNA in liver and heart from rats [12, 13], further impairing mitochondrial function and leading to a vicious circle in which ROS production continues to increase [12–14]. Conversely, biogenesis of mitochondria is associated with an increased survival of septic shock [11], and experimental models of sepsis-AKI confirm the importance of maintaining healthy mitochondria for renal recovery and survival [15–19].

To date, the precise molecular mechanisms of mitochondria in the pathogenesis of sepsis-AKI are incompletely understood, which hampers the development of a molecular targeted therapy to prevent or treat sepsis-AKI and improve outcome. The high metabolic burden during sepsis leads to increased ROS production by mitochondria. We hypothesized that damage to mitochondrial DNA during sepsis causes mitochondrial dysfunction with relevance to both short-term outcome and potentially also for long-term outcome in sepsis-AKI. We therefore explored the mRNA expression of mitochondrial quality mechanism pathways and studied mitochondrial DNA (mtDNA) oxidation and integrity in renal biopsies from sepsis-AKI patients and control subjects. Next, we validated these results in vitro, by inducing human umbilical vein endothelial cells (HUVECs) with lipopolysaccharide (LPS) for 48 h to mimic sepsis.

## Material and methods

### Patients

We collected postmortem renal biopsies from patients as quickly as possible after death [24–150-min postmortem] from fourteen patients who died of sepsis with acute kidney injury at the ICU of the University Medical Center Groningen (UMCG). The biopsy was taken from the renal cortex, and once removed from the patient, the biopsies were immediately snap frozen using liquid nitrogen and stored in the − 80 °C until analysis. No in vitro perfusion took place. Due to the rapid sampling time and immediate freezing, we eliminated unwanted tissue necrosis and autolysis, which would disturb the analysis. Patients were defined as having septic shock according to the internal sepsis definitions, comprising sepsis with atrial hypotension despite adequate fluid resuscitation [20], while AKI was classified according to the RIFLE criteria [21]. Severity of illness was defined upon admission to the ICU using the Acute Physiology and Chronic Health Evaluation (APACHE) IV score and the Simplified Acute Physiology Score (SAPS) II score [22, 23]. Informed written consent for performing biopsies was obtained during the family meeting, before or just after death. In control subjects, following preoperative consent, non-septic kidney biopsies were obtained from twelve patients who underwent complete nephrectomy due to renal cell carcinoma. In this procedure, a healthy section of the kidney cortex was taken, as far away as possible from the carcinoma. Nephrectomy biopsies were analyzed by a pathologist and considered to be normal healthy controls. Additional details of the collection of kidney biopsies are described elsewhere [24]. Both septic patients and controls were 18 years or older. Patients with preexisting chronic kidney disease (CKD), active autoimmune disorders with renal involvement and treatment with immune-suppressive medication were excluded from this study. The Medical Ethics Review Committee (METC) of the UMCG reviewed and approved this study (METC 2011/372). Patient characteristics and clinical and laboratory details can be found in Table 1.

**Table 1:** Patient characteristics

	Control (*n* = 12)	Sepsis-AKI (*n* = 14)
Mean age (years)	62 (20–79)	76 (53–85)
Sex (male:female)	5:7	11:3
Comorbidities/medical history (*n*)		
Hypertension	3	6
Diabetes mellitus	1	1
COPD or asthma	4	2
Coronary artery disease	1	5
Renal disease	0	0
Auto-immune disease	0	1
Neoplasms (extra-renal)	4	1
RIFLE stage (*n*)	N/A	
Risk		0
Injury		6
Failure		8
Lost renal function		0
End-stage kidney failure		0
Need for RRT in ICU: yes/no	N/A	5/14
Serum creatinine at admission (μmol/L)	N/A	135 (81–355)
Serum creatinine before biopsy (μmol/L)	73 (58–114)	156 (73–401)
APACHE IV score	N/A	97 (50–175)
SAPS II score	N/A	65 (35–88)
Sepsis focus	N/A	
Lower respiratory tract		5
Skin/soft tissue		2
Intra-abdominal (Mesenteric ischemia, necrotizing pancreatitis)		6
Endovascular (endocarditis)		1

Data are presented as median with upper and lower range. *N/A* not applicable, *RRT* renal replacement therapy, *ICU* intensive care unit, *APACHE* Acute Physiology and Chronic Health Evaluation, *SAPS* Simplified Acute Physiology Score

### Cells

Human umbilical vein endothelial cells (HUVECs) and medium were obtained from the UMCG Endothelial Cell Facility. Briefly, primary isolates of umbilical cords were mixed and subsequently cultured on HUVEC culture medium, consisting of RPMI 1640 (Lonza, Breda, Netherlands) supplemented with 20% heat-inactivated fetal calf serum (ThermoFisher, Waltham, MA), 2 mM l-glutamine (Life Technologies, Carlsbad, CA), 5 U/ml heparin (Leo Pharmaceutical Products, The Netherlands), 1% Penicillin/Streptomycin (Sigma-Aldrich, St. Louis, MI) and 50 μg/ml EC growth factor supplement from (Sigma-Aldrich). Cells were plated in 6-well culture plates (Corning, St. Louis, MI), and at 80% confluency cells were stimulated with 10 µg/ml lipopolysaccharide (LPS) *E. coli* 0111:B4 (Sigma-Aldrich) for 48 h.

### DNA isolation

Total DNA was isolated to perform a polymerase chain reaction (PCR) and determine mitochondrial DNA damage. DNA was isolated from renal biopsies of eight controls subjects and twelve sepsis-AKI patients. First, sections of 5 μm thickness were cut from the renal biopsies. Samples were pretreated with 500 µL collagenase V (300 U/mL; Sigma-Aldrich, Darmstadt, Germany), incubated at 37 °C for 3 h and vortexed every 30 min. Subsequently, 500 µL RPMI (ThermoFisher, Paisly, UK) was added, followed by centrifugation for 10 min at 20,000 G at room temperature. Next, Rapid Sample Concentrator (RSC) blood DNA kit (Promega, Madison, USA) was used to isolate DNA from the pellet using Maxwell 16 MDx AS3000 (Promega), according to the manufacturer’s protocol. DNA from HUVECs was isolated with Nucleospin DNA kit (MACHEREY–NAGEL, GmbH & Co. KG, Germany), according to manufacturer’s protocol.

### Quantification of mitochondrial mass and DNA damage

Mitochondrial copy number, indicative of mitochondrial mass, and mitochondrial DNA damage were determined using quantitative polymerase chain reaction (qPCR). Oligonucleotide primers (Sigma-Aldrich; Table 2) were designed using Clone Manager 9 software and validated by assessing the efficiency, melting- and temperature curves using CFX384- Real-Time system (Biorad, California, USA). Amplification of the DNA was performed using the following thermal profile: 95 °C for 2 min, followed by 40 cycles of 95 °C for 15 s, 60 °C for 30 s and 72 °C for 30 s. All reactions were carried out in duplicate, and the obtained threshold cycle (Ct) values were averaged. MtDNA copy number was calculated using the average levels of the mitochondrial genes: NADH dehydrogenase 1 (*ND1*), NADH dehydrogenase 4 (*ND4*), NADH dehydrogenase 6 (*ND6*), Cytochrome C Oxidase I (*COX1*), Cytochrome B (*CYTB*) and *D-loop*, divided by the amount of nuclear housekeeping gene Beta 2-microglobulin (*B2M*)*,* using the 2 − ∆∆Ct method.

**Table 2:** List of primers used for quantification of mRNA and DNA expression levels by qPCR and RT-qPCR

Gene	Primer sequence	
*ND1*, ETC complex 1	Forward:	TGGCTCCTTTAACCTCTCCA
	Reverse:	GGTTCGGTTGGTCTCTGCTA
*ND4*, ETC complex 1	Forward:	GGCGGCTATGGTATAATACG
	Reverse:	GTAGGCAGATGGAGCTTGTT
*ND6*, ETC complex 1	Forward:	TGATTGTTAGCGGTGTGGTC
	Reverse:	CCTCAATAGCCATCGCTGTA
*COX1*, ETC complex 4	Forward:	CTAACAGACCGCAACCTCAA
	Reverse:	CCGAAGCCTGGTAGGATAA
*Cytochrome b*, ETC complex 3	Forward:	TTCCTAGCCATGCACTACTC
	Reverse:	GAAGGTAGCGGATGATTCAG
*D-loop*, regulative region	Forward:	AACCTACCCACCCTTAACAG
	Reverse:	CACTCTTGTGCGGGATATTG
*β2M*	Forward:	CTGGGTAGCTCTAAACAATGTATTCA
	Reverse:	CATGTACTAACAAATGTCTAAAATGGT

*ETC* electron transport chain

Mitochondrial DNA damage was assessed by qPCR for determination of a short (~ 200 bp) mtDNA part and long-range PCR, for determination of a long (10 kb) mtDNA part. First, the relative amount of each mitochondrial gene was quantified by qPCR and divided by the amount of nuclear housekeeping gene *B2M,* using the 2 − ∆∆Ct method. Next, a long-range PCR was performed using the TaKaRa LA Taq DNA polymerase kit (Takara Bio, Kusatsu, Japan) to amplify a 10-kb mtDNA template, stretching from the *ND5* to *ND1* gene, thereby comprising more than two-third of the mitochondrial genome. A short mtDNA fragment of 222 bp was amplified by qPCR to serve as reference (Table 3). The long fragment was amplified with T100 Thermal Cycler (Biorad, California, USA), using the following thermal profile: 94 °C for 1 min, followed by 18 cycles of 15 s at 94 °C and 12 min at 64 °C, and ending with 10 min at 72 °C. The short fragment was amplified using the following thermal profile: 95 °C for 2 min, followed by 40 cycles of 95 °C for 15 s and 60 °C for 30 s. Both PCR products were separated and visualized on a 1% agarose gel (45 min, 100 V), and their intensity was analyzed using ImageJ [25]. The ratio of the short to long fragment was calculated to quantify mtDNA damage. Due to limited sample material, DNA from six control subjects and twelve sepsis-AKI patients was used for long-range PCR, whereas material from seven control subjects and twelve sepsis-AKI patients was used for qPCR.

**Table 3:** List of primers used for amplification of mitochondrial DNA with long-range PCR and qPCR

Primer	Primer sequence
Forward long fragment mtDNA (10 kb)	TCTAAGCCTCCTTATTCGAGCCGA
Forward Short fragment mtDNA (222 bp)	CCCCACAAACCCCATTACTAAACCCA
Reverse primer mtDNA (short and long)	TTTCATCATGCGGAGATGTTGGATGG

*mtDNA* mitochondrial DNA

### RNA isolation and quantification of gene expression

First, total RNA was isolated from twelve control subjects and twelve sepsis-AKI patients for reverse transcription by quantitative polymerase chain reaction (RT-qPCR). RNA was isolated from 20 × 5 µm kidney cryosections using the RNeasy Mini Plus Kit (Qiagen, Leusden, The Netherlands), according to the manufacturer's protocol. RNA integrity was determined by gel electrophoresis and consistently found intact. RNA yield and purity were measured by an ND-1000 UV–Vis spectrophotometer (NanoDrop Technologies, Rockland, DE). cDNA was synthesized as previously described [26]. RNA from HUVECs was isolated using Nucleospin RNA kit (MACHEREY–NAGEL). Cells were pretreated with TRIzol, followed by RNA isolation according to manufacturer’s protocol. Next, oligonucleotide primers (Sigma-Aldrich; Table 4) were designed using Clone Manager 9 software and validated by assessing the efficiency, melting- and temperature curve using RT-qPCR. RT-qPCR amplification was performed using the following thermal profile: 95 °C for 2 min, followed by 40 cycles of 95 °C for 15 s, 60 °C for 30 s and 72 °C 30 s. Reactions were carried out in duplicate, and the obtained Ct values were averaged. Gene expression (mRNA) was normalized using *β-actin* as a housekeeping gene, and 2 − ∆∆Ct was used to obtain relative quantity.

**Table 4:** List of primers for quantification of mRNA expression of mitochondrial quality mechanisms, mitochondrial complexes and oxidation pathways by RT-qPCR

Gene	Primer sequence		Process
*PINK1*	Forward:	GACGCTGTTCCTCGTTATGA	Mitophagy
	Reverse:	TCCAGCTCCACAAGGATGTT	
*PARKIN*	Forward:	GACCCTCAACTTGGCTACTC	Mitophagy
	Reverse:	CTTCGCAGGTGACTTTCCTC	
*MFN2*	Forward:	CCGCCACATAGAGGAAGGAC	Fusion
	Reverse:	CGCACAGACACAGGAAGGAG	
*DRP1*	Forward:	AAGCTGCTGCCATAGTCCTC	Fission
	Reverse:	ACCACAGCCATGTCAGTGTC	
*NRF2*	Forward:	GCTACTAATCAGGCTCAGTC	Mitochondrial biogenesis
	Reverse:	GTAGTCTCAACCAGCTTGTC	
*TFAM*	Forward:	CATGGACTTCTGCCAGCATA	Mitochondrial biogenesis
	Reverse:	AGAACACCGTGGCTTCTACA	
*mnSOD*	Forward:	ACGCGGCCTACGTGAACAAC	Antioxidant enzyme
	Reverse:	CAACAGATGCAGCCGTCAGC	
*OGG1*	Forward:	GTGTGCGACTGCTGCGACAA	mtDNA damage repair (excision of 8-oxoguanine)
	Reverse:	CTGGATGAGCCGAGGTCCAA	
*HIF1α*	Forward:	TGAGGGGACAGGAGGATCAG	Master regulator of cellular and systemic homeostatic response to hypoxia
	Reverse:	CACGCGGAGAAGAGAAGGAA	
*SIRT1*	Forward:	ATGCTGGCCTAATAGAGTGG	Regulates epigenetic gene silencing, biogenese and antioxidant mechanisms
	Reverse:	TCTGGAACATCAGGCTCATC	
*NGAL*	Forward:	GGTGAGCACCAACTACAACC	Early biomarker of acute kidney injury
	Reverse:	GCCCAGAGATTTGGAGAAGC	
*NDUFA1*	Forward;	ACTGGCTACTGCGTACATCC	ETC complex 1 (nDNA)
	Reverse:	AGATGCGCCTATCTCTTTCC	
*COX5b*	Forward:	CATTGGCTCCTTCTCCCATA	ETC complex 4 (nDNA)
	Reverse:	CATACCAGGTGGTCCCATTC	
*B-actin*	Forward:	AGGATGCAGAAGGAGATCAC	Housekeeping gene
	Reverse:	AGTCATAGTCCGCCTAGAAG	

*mtDNA* mitochondrial DNA, *ETC* electron transport chain, *nDNA* nuclear DNA

### Immunohistochemical analysis

DNA oxidation was assessed by immunohistochemical analysis of 8-oxoguanine (8-oxoG) on formalin-fixed paraffin-embedded kidney sections. Therefore, sections were deparaffinized in xylene and rehydrated in graded ethanol series (70–100%) and distilled water. After deparaffination, heat-induced epitope retrieval was performed using citrate buffer (pH 6) antigen retrieval. Endogenous peroxidase activity was blocked by incubating the slides with 3% hydrogen peroxide. After washing, the slides were incubated with anti-human 8-oxoG Antibody, (Abcam, Cambridge, UK) diluted 1:400 in antibody solution (5% fetal calf serum in PBS) for 1 h at room temperature. After washing, slides were incubated with rabbit anti-mouse IgG secondary antibody (Southern Biotech, Birmingham, USA) diluted 1:100 in antibody solution with 2% normal human serum (NHS) for 45 min at room temperature. Slides were washed and incubated with anti-rabbit horse radish peroxidase-labeled antibody (EnVision kit, DAKO Cytomation, Glostrup, Denmark). Peroxidase activity was detected using 3-amino-9-ethylcarbazole (AEC) complex, and the sections were subsequently counterstained with Mayer’s hematoxylin (Merck, Darmstadt, Germany) before mounting in Aquatex mounting agent (Merck). 8-oxoG staining was visualized with a Leica DCF295 color camera (Leica, Heerbrugg, Switzerland), and images taken with the Leica software application suite (LAS version 4, Leica).

### Immunofluorescent analysis

Co-localization of DNA oxidation and mitochondria was assessed by immunofluorescent analysis of Translocase Of Outer Mitochondrial Membrane (TOM20) (mitochondrial immunolabeling) and 8-oxoG on kidney cryosections (9 µm). In short, the slides were air-dried, fixated with ice-cold acetone for 10 min, washed with PBS and permeabilized with 0.25% Triton-X-100 for 10 min. Then, sections were incubated for 1 h at room temperature with the primary 8-oxoG antibody (Novus Biologicals, Abingdon, Oxon), diluted 1:75 in 1% bovine serum albumin (BSA) in PBS, washed with PBS and incubated for 30 min with Donkey anti-Goat secondary antibody (ThermoFisher), diluted 1:100 in 1% PBS/BSA. Next, slides were incubated for 1 h at room temperature with TOM20 primary antibody (Santa Cruz, Dallas, USA), diluted 1:20 in 1% PBS/BSA, followed by washing with PBS and incubation for 30 min with Donkey anti-Rabbit IgG secondary antibody (ThermoFisher), diluted 1:100 in 1% PBS/BSA. Slides were mounted in Vectashield with DAPI (Vector Laboratories Inc., Burlingame, CA, USA) and visualized with a Leica DM2000 microscope (Leica, Amsterdam, Netherlands). Co-localization was identified using ImageJ software.

### Statistical analysis and data presentation

Statistical analysis was performed with IBM SPSS 23.0 for Windows (IBM Corp., Armonk, N.Y., USA) and GraphPad Prism Software version 7.02 (GraphPad Prism software Inc., San Diego, CA, USA). Two-tailed Mann–Whitney *U* tests were used to calculate statistical differences between groups (using GraphPad Prism software), whereas correlations between selected groups were assessed by Spearman’s Rho tests in IBM SPSS. *P* < 0.05 was considered significant different. Data are expressed as median with upper and lower ranges. Figures were made with GraphPad Prism (GraphPad Prism software Inc.), bars represent the median and each dot represents an individual.

## Results

### Study population

**Fig. 1:**
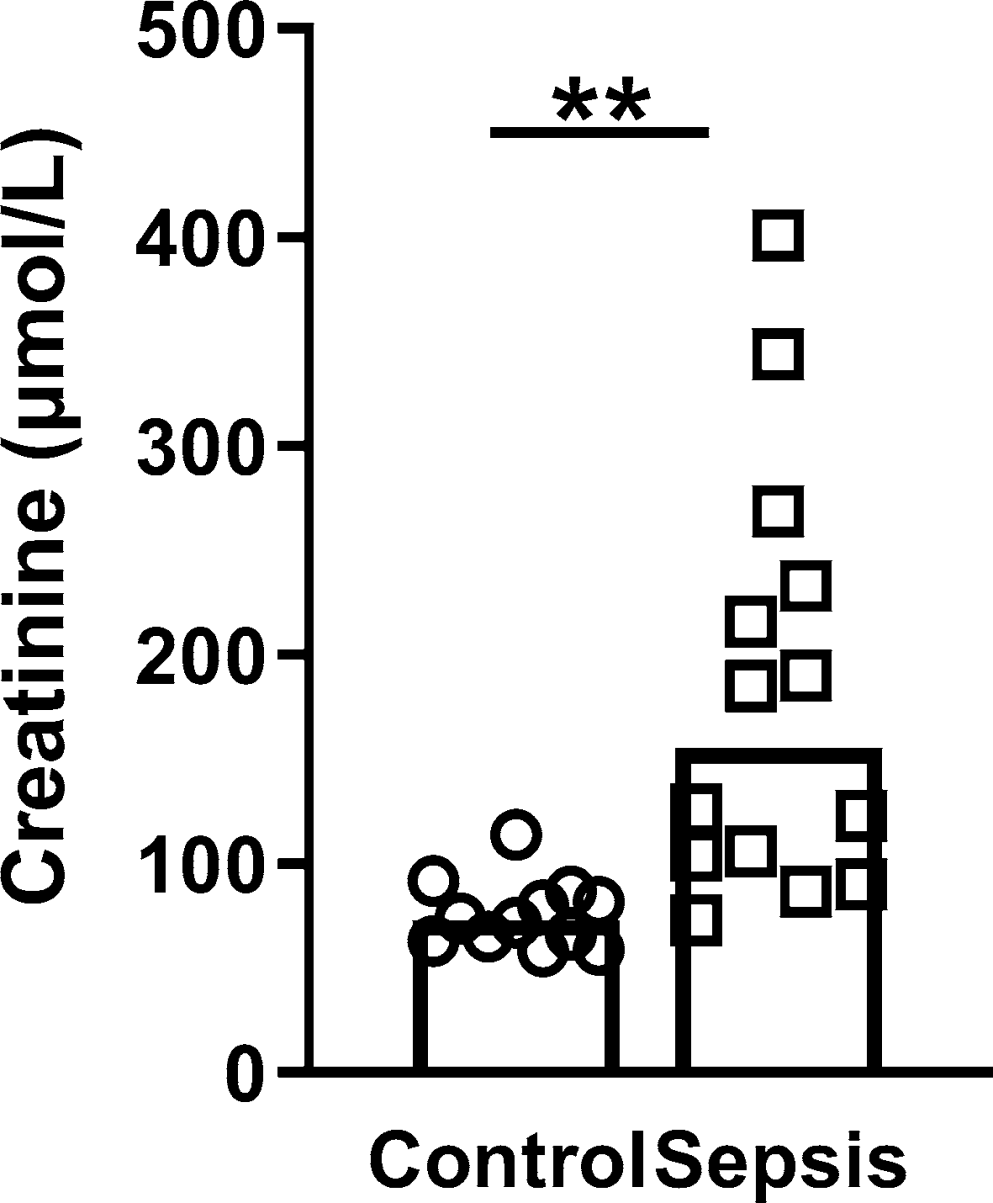
Serum creatinine concentrations. Serum concentrations of creatinine (µmol/L) in control subjects and sepsis-AKI patients before nephrectomy or prior to extirpation, respectively

Renal biopsies from fourteen patients with sepsis-AKI and twelve control subjects were examined. Six sepsis-AKI patients had an intra-abdominal infection, five patients suffered from a lower respiratory tract infection, two patients had a skin/soft tissue infection and one patient suffered from endocarditis. The median age of patients with sepsis-AKI was 76 (range 53–85) years, and for control subjects the median age was 62 (range 20–79) (Table 1). The male-to-female ratio was 11:3 and 5:7 for sepsis-AKI and control subjects, respectively. Six sepsis-AKI patients were categorized in the Injury stage of the RIFLE criteria and eight in the Failure stage. Five patients needed renal replacement therapy in the ICU. Clinical characteristics are listed in Table 1, and serum creatinine before nephrectomy in control subjects or before the biopsy was taken in sepsis-AKI patients is shown in Fig. 1.

### Sepsis-AKI is associated with renal oxidative stress

**Fig. 2:**
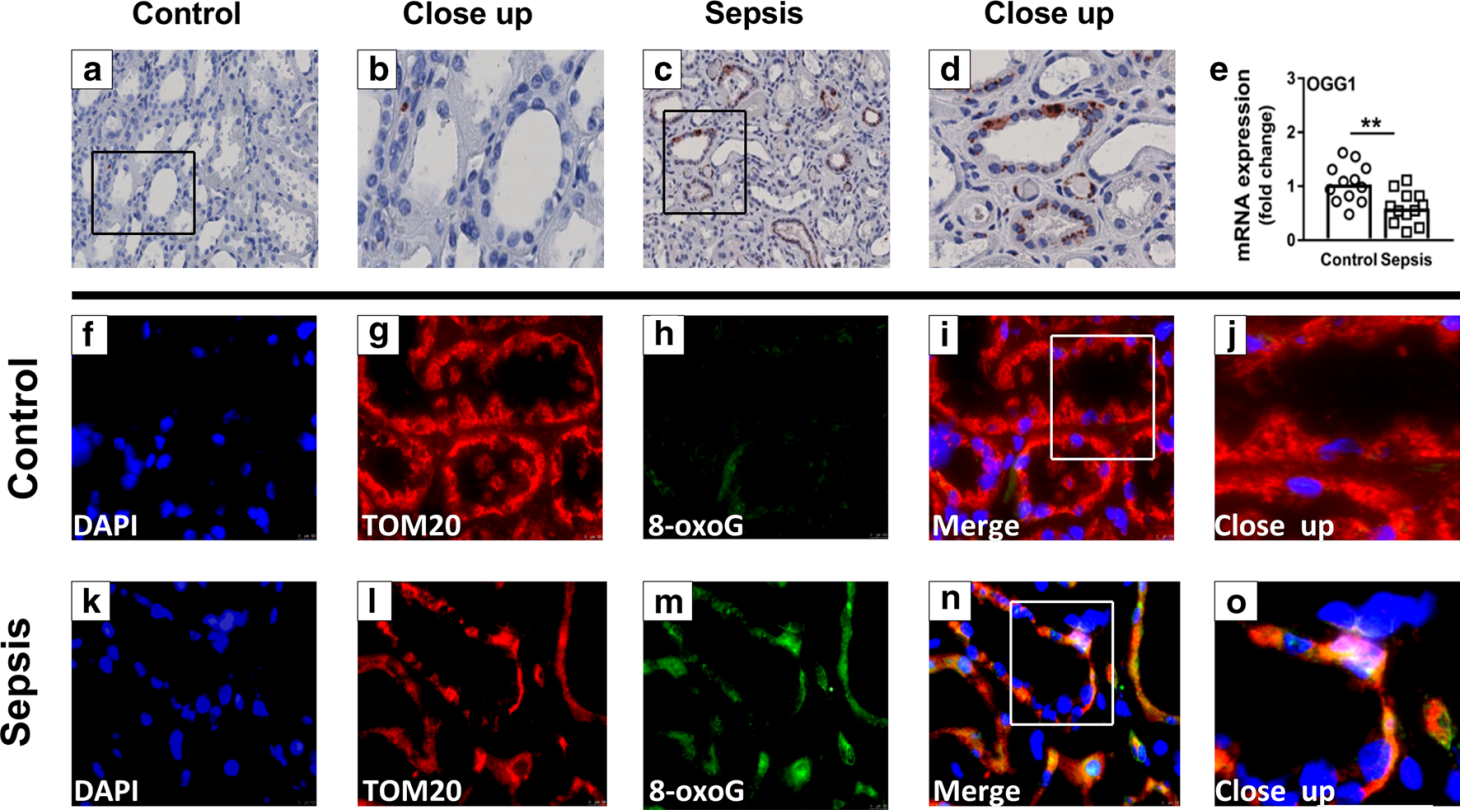
Oxidative DNA damage in controls and sepsis-AKI subjects. **a**–**d** Immunohistochemistry of 8-oxoG (DNA oxidation marker) **e** mRNA expression of *OGG1* as quantified by RT-qPCR **f**–**o** immunohistochemistry of TOM20 (mitochondrial marker) and 8-oxoG (DNA oxidation marker). Bars represent median, dots represent individual levels; ** means *p* < 0.005

To explore whether sepsis-AKI leads to oxidation of DNA, we performed an 8-oxoG staining and quantified the expression of the base-excision repair enzyme *8-oxoguanine DNA glycosylase* (*OGG1*) in kidney biopsy material. 8-oxoG staining, a mutagenic base byproduct that occurs as a result of exposure to reactive oxygen species, was present in some of the tubular epithelial cells of all sepsis-AKI patients, but almost absent in biopsies from control subjects (Fig. 2a–d). Expression of *OGG1*, the primary enzyme responsible for the excision of 8-oxoG, was downregulated in patients with sepsis (*p* < 0.005; Fig. 2e). Fluorescent staining of the mitochondrial marker TOM20 and 8-oxoG demonstrated their colocalization, implicating oxidation of the mitochondrial DNA (Fig. 2j, o).

**Fig. 3:**
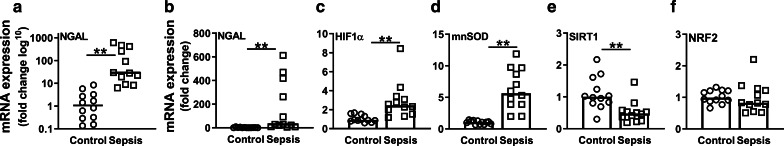
Gene expression of oxidative damage- and antioxidant markers. mRNA expression of **a** *NGAL* (log^10^), **b** *NGAL*, **c** *HIF1α*, **d** *mnSOD*, **e** *SIRT1*, and **f*** NRF2* as quantified by RT-qPCR*.* Bars represent median, dots represent individual levels; ** means *p* < 0.005

**Fig. 4:**
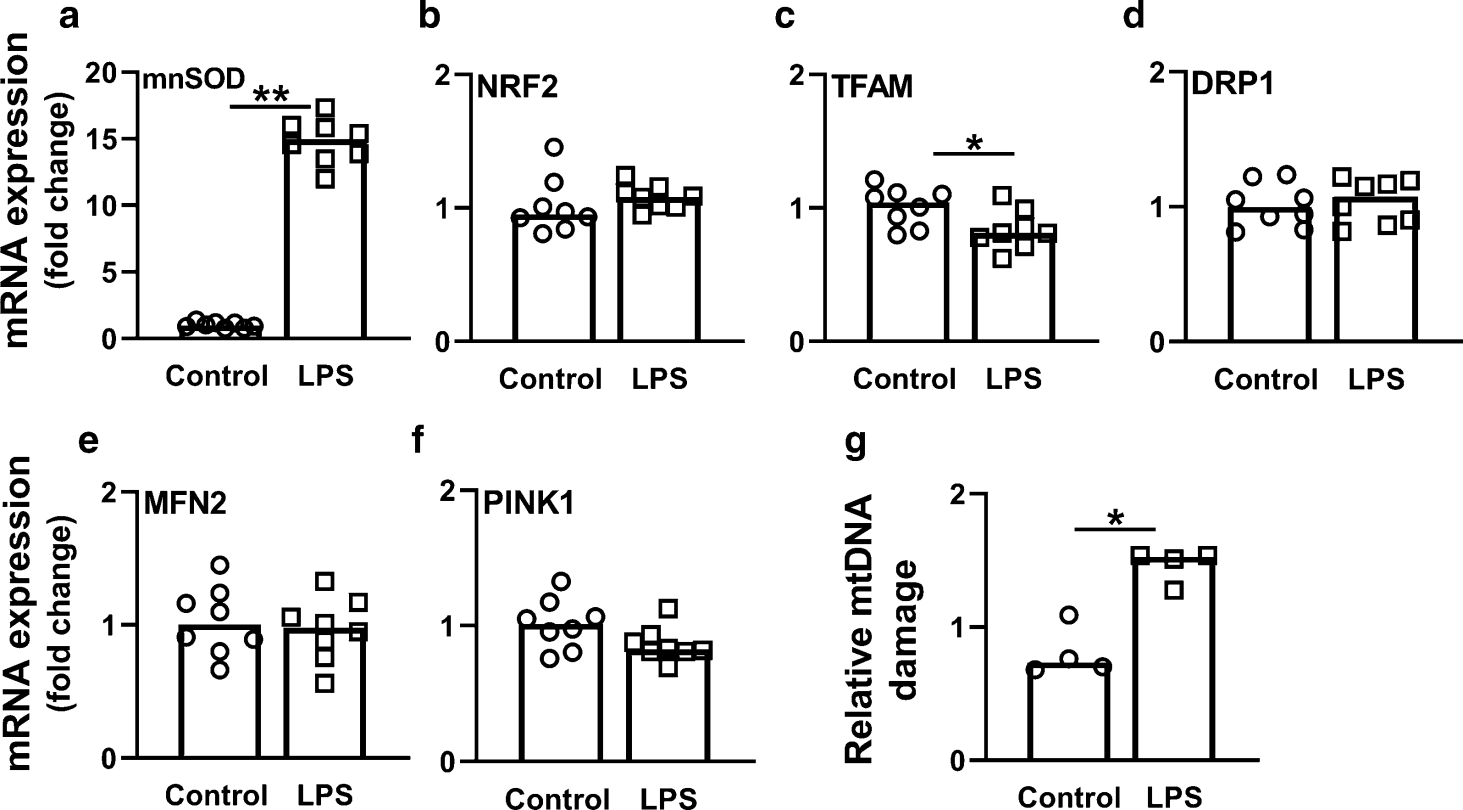
Changes in mitochondrial biogenesis, fusion, fission and mitophagy after 48-h LPS induction in HUVECs. Cells were induced with 10 µg/mL LPS for 48 h. mRNA expression of **a** *mnSOD*, **b** *NRF2*, **c** *TFAM*, **d** *DRP1*, **e** *MFN2*, and **f** *PINK1*; **g** Relative mtDNA damage levels of control and LPS-induced HUVECs. Bars represent median, dots represent individual levels; * means *p* < 0.05 and ** *p *<0.005

To confirm the presence of kidney damage in sepsis-AKI patients, we quantified mRNA expression of neutrophil gelatinase-associated lipocalin (*NGAL*), a marker for kidney damage and oxidative stress [26]. Septic patients showed a significantly upregulation of *NGAL* (*p* < 0.001; Fig. 3a, b). In addition, sepsis-AKI patients showed an increased expression of hypoxia-inducible factor 1-alpha (*HIF1α*), the master oxygen sensor within cells and manganese superoxide dismutase (*mnSOD*), a key antioxidant enzyme, suggesting an active response to oxidative stress (both *p* < 0.001; Fig. 3c, d). *mnSOD* positively correlated with *NGAL* and *HIFα* (*R* = 0.858 and *R* = 0.921, respectively, both *p* < 0.001). In contrast, sepsis-AKI patients had lowered mRNA expression of Sirtuin 1 (*SIRT1*), involved in inhibiting oxidative stress and in biogenesis, as compared to control subjects (*p* < 0.01; Fig. 3e), while *NRF2* important in regulation of antioxidant protein expression was not different between both groups (Fig. 3f). Also in HUVECs the *mnSOD* expression was significantly increased after 48 h of LPS induction compared to controls (*p* < 0.001, Fig 4a), while *NRF2* was not different (Fig. 4b). Taken together, patients with sepsis-AKI likely display increased levels of oxidative stress, as demonstrated by increased levels of 8-oxoG in tubular cells and increased mRNA expression of oxidative stress defense pathways.

### Sepsis-AKI is associated with mitochondrial DNA damage in the kidney

**Fig. 5:**
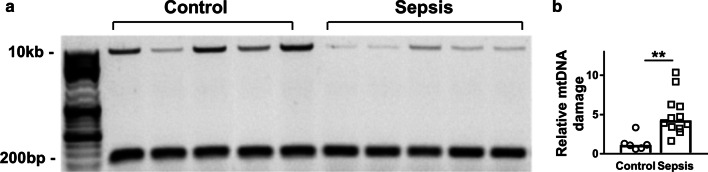
Mitochondrial DNA damage in control and sepsis-AKI subjects. A 10-kb-long mtDNA sequence was amplified by long-range PCR, while a 222-bp short mtDNA sequence was amplified with qPCR. **a** Amplified mtDNA sequences were loaded on agarose gel from representative samples of control subjects and sepsis-AKI subjects; **b** relative mtDNA damage of the control subjects compared to the sepsis-AKI patients. Bars represent median, dots represent individual levels; ** means *p* < 0.005

**Fig. 6:**
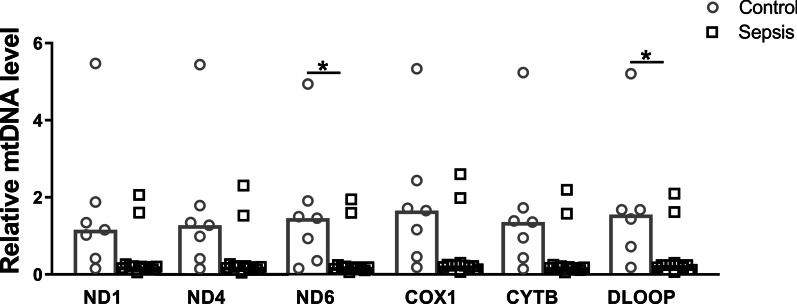
Relative mitochondrial DNA levels. Relative mtDNA levels of *ND1*, *ND4, ND6, COX1, CYTB,* and *D-loop* as quantified by qPCR. Bars represent the median, dots represent individual levels; * means *p* < 0.05

The integrity of mtDNA is critical to maintain normal mitochondrial function [27]. To investigate whether sepsis-AKI alters the integrity of mtDNA, we measured mtDNA damage by long-range PCR and quantified the ratio between a long and short mtDNA fragment, with higher ratios denoting more mtDNA damage (Fig. 5a). Kidney mtDNA damage was higher in sepsis-AKI patients as compared to patients without sepsis-AKI (*p* < 0.01, Fig. 5b). Levels of mtDNA damage positively correlated with expression of *mnSOD* and *HIF1α*, markers for oxidative stress, and renal damage marker *NGAL* (*R* = 0.77, *R* = 0.68 and *R* = 0.89, respectively, all *p* < 0.01) and negatively correlated with base-excision repair enzyme *OGG1* and biogenesis marker mitochondrial transcription factor A (*TFAM)* (*R* = − 0.51 and *R* = − 0.65, respectively, both *p* < 0.05). However, mtDNA damage did not correlate with the severity of critical illness as defined by the APACHE IV and SAPSII scores. Furthermore, sepsis-AKI patients had lower DNA levels of mitochondrial genes *ND6*, and *D-loop*, whereas DNA levels of *ND1*, *ND4*, *COX1* and *CYTB* were not different between control subjects and patients with sepsis-AKI, probably due to the small sample size and thus a type 2 error (Fig. 6). Next, we explored the mtDNA damage in 48-h LPS-induced HUVECs. In keeping, we found an increase in mtDNA damage after 48 h of LPS induction (Fig. 4g). Collectively, these findings show that sepsis is associated with mtDNA damage.

### Mitochondrial quality control does not compensate for mtDNA damage

**Fig. 7:**
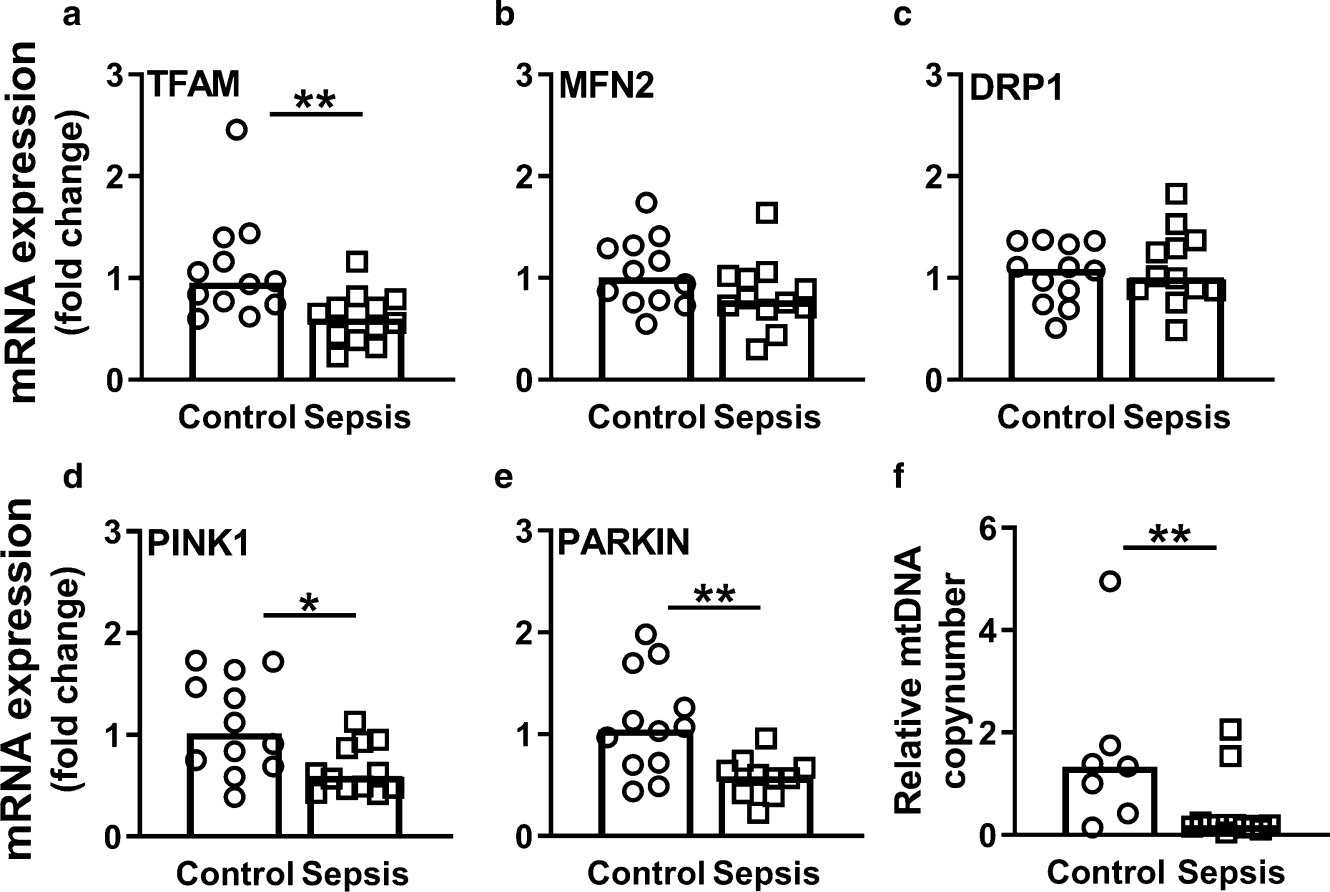
Changes in mitochondrial biogenesis, fusion, fission, mitophagy and copy number in response to sepsis-AKI. mRNA expression of **a** *TFAM*, **b** *MFN2*, **c** *DRP1*, **d** *PINK1,* and **e** *PARKIN as* quantified by RT-qPCR; **f** mtDNA levels were quantified by RT-qPCR to calculate the mitochondrial copy number. Bars represent median, dots represent individual levels; */** means *p* < 0.05/0.005; *mtDNA* mitochondrial DNA

**Fig. 8:**
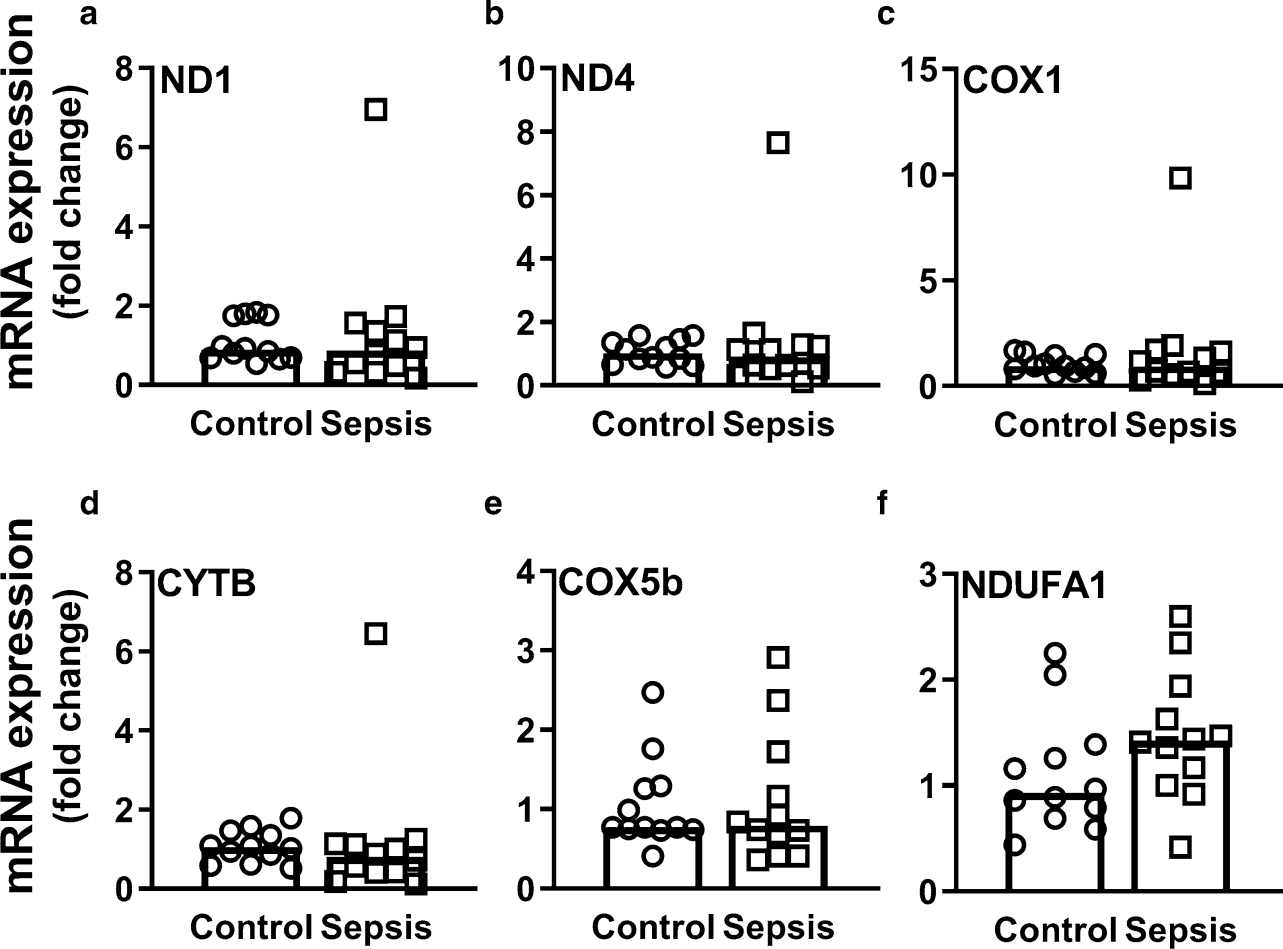
mRNA expression of mitochondrial electron transport chain complexes. mRNA expression of **a** *ND1*, **b** *ND4*, **c** *COX1*, **d** *CYTB*, **e** *COX5b,* and **f** *NDUFA1* as quantified by RT-qPCR. Bars represent median, dots represent individual levels

To assess mitochondrial quality control, which is important to maintain a healthy mitochondrial pool and consist of biogenesis, mitophagy, fission and fusion [28], we measured mRNA expression of genes involved in these processes. Expression of the biogenesis marker *TFAM* was lower in sepsis-AKI patients than in control subjects (*p* < 0.01; Fig. 7a). Although sepsis-AKI led to reduced mtDNA levels and integrity, it did not affect mRNA expression of mitochondrial genes encoding key components of the mitochondrial electron transport chain complexes (*i.e., ND1*, *ND4*, *COX1*, *CYTB*, *COX5b* and *NDUFA1*) (Fig. 8). Further, sepsis-AKI did not affect mRNA expression of the mitochondrial fusion and fission markers mitofusin 2 (*MFN2*) and dynamin-related protein 1 (*DRP1*), respectively (Fig. 7b, c). As expected, however, *MFN2* and *DRP1* did correlate with each other (*R* = 0.58, *p* < 0.01). Sepsis-AKI patients had lower mRNA expression of PTEN-induced putative kinase 1 (*PINK1*) and *PARKIN*, key components in regulating mitophagy and therefore important in the removal of unhealthy mitochondria, as compared to control subjects (*p* < 0.05 and *p* < 0.01, respectively; Fig. 7d, e). Expression of *PINK1* correlated with *PARKIN* expression (*R* = 0.56, *p* < 0.01). Accordingly, 48-h LPS induction in HUVECS caused a decrease in *TFAM* mRNA expression, but did not change *MFN2*, *DRP1* or *PINK1* expression (Fig. 4c–f). Collectively, these data imply that sepsis-AKI patients and LPS-induced HUVECs did not have a compensatory increase in mitochondrial quality control mechanisms.

### Sepsis-AKI causes a decrease in mitochondrial mass

Based on the increase in antioxidant markers and decrease in mitochondrial biogenesis markers, we hypothesized that patients with sepsis-AKI had a reduction in the mitochondrial mass, due to increased oxidative stress while having reduced mitochondrial quality control. To estimate the mitochondrial mass, we determined the mitochondrial copy number by calculating the ratio between the expression of mitochondrial genes and the nuclear housekeeping gene *B2M*. In keeping with the elevated mitochondrial DNA damage ratio, mitochondrial DNA levels were reduced in sepsis-AKI compared to control subjects, denoting a decrease in mitochondrial mass (*p* < 0.05; Fig. 7f). Hence, sepsis-AKI shows increased mitochondrial DNA damage ratio, a decreased mitochondrial mass, in the absence of compensation of mitochondrial quality control markers.

## Discussion

Sepsis leads to increased levels of ROS, which is in part due to the overwhelming immune response to facilitate bacterial killing [1, 27, 29]. In turn, ROS can cause collateral damage by oxidizing proteins, lipids and DNA [12, 13]. Subsequent damage to mtDNA can impair mitochondrial function and further increase ROS generation [27, 29]. Whether this process underlies the pathophysiology in sepsis-AKI was not yet known. Here, we demonstrate that sepsis-AKI patients have an upregulated mRNA expression of markers important in the renal oxidative stress response, leading to higher levels of mtDNA oxidation, mtDNA damage and a reduced number of mitochondria in the kidney. Despite the mitochondrial damage, we did not find evidence of compensatory upregulation of mitochondrial quality control mechanisms, which further substantiates mitochondrial dysfunction in sepsis-AKI. Together, these data demonstrate key mechanisms leading to mitochondrial failure in the pathophysiology of sepsis-AKI.

The way cells deal with ROS under inflammatory circumstances is important for the distinction between survival, long-term complications, or mortality of patients with sepsis [30]. Induction of *NGAL, mnSOD* and *HIF1α* is indicative of renal oxidative stress and injury [31–33]. One of the first markers of renal injury is upregulation of *NGAL* expression [34], as demonstrated in our biopsies derived from patients with sepsis-AKI. Increased *NGAL* expression is regulated by ROS to suppress bacterial growth and modulate the inflammatory response [31]. In turn, *NGAL* activates antioxidant defense mechanisms, leading to the upregulation of *mnSOD*, as demonstrated in vitro [31]. Accordingly, we found a positive correlation between *NGAL* expression and *mnSOD* expression in the renal biopsies. The importance of upregulated *mnSOD* in sepsis is confirmed in mouse models of sepsis (i.e., lipopolysaccharide [LPS] injection and cecal ligature and puncture [CLP]), where higher *mnSOD* expression prevented ATP depletion and subsequent mortality [33, 35, 36]. Similarly, we found an increased gene expression of *mnSOD* expression in patients with sepsis-AKI as compared to control subjects. Upregulation of *HIF1α* switches mitochondrial aerobic respiration to glycolysis metabolism, thereby bypassing the dysfunctional ROS-producing mitochondria and lowering oxidative stress [32]. Higher *HIF1α* expression levels were detected in whole blood cells from patients with septic shock [37], in line with the increased expression in the kidney as demonstrated here in sepsis-AKI. In contrast to *NGAL*, *mnSOD* and *HIF1α*, the expression of *SIRT1*, involved in inhibiting oxidative stress and the suppression of biogenesis and mitophagy [38, 39], was downregulated in sepsis-AKI patients. Taken together, sepsis-AKI is associated with upregulation of genes encoding molecules involved in inhibiting inflammation and antioxidant defense in the kidney.

Compared to control subjects, sepsis-AKI patients had upregulated mRNA expression of oxidative damage markers and high levels of mitochondrial DNA damage. Also 48 h of LPS induction in HUVECs caused an increase in *mnSOD* expression and mtDNA damage. Although several studies demonstrated increased biomarkers suggestive of mitochondrial dysfunction in sepsis [10, 11, 15], to our knowledge only one other study so far demonstrated the occurrence of mtDNA damage in patients with sepsis, as illustrated by the depletion of mtDNA defined by RT-qPCR in monocytes and lymphocytes [40]. Extending on the observations of these previous studies, we now directly demonstrate the presence of mtDNA damage in the septic kidney. Whereas mtDNA damage in monocytes and lymphocytes correlated with the APACHE score, we did not find such a correlation between mtDNA damage and APACHE score in sepsis-AKI patients, which might be due to the small sample size (*n* = 147 vs *n* = 12, respectively). The observed mtDNA damage in the kidney in patients with sepsis-AKI is in line with findings from experimental murine sepsis models, which showed profound damage to mtDNA in skeletal muscle and 50% mtDNA depletion in the liver [9, 41]. mtDNA damage was associated with increased DNA oxidation (i.e., 8-oxoG) in sepsis-AKI as compared to control subjects, while immunofluorescent staining showed co-localization of DNA oxidation with both nuclei and mitochondria. In line with this observation, sepsis in mice also leads to accumulation of 8-oxoG in mitochondria and mitochondrial dysfunction, as demonstrated in the brain [42]. Hence, mitochondrial dysfunction leading to increased oxidative stress, oxidation of mtDNA and damage that further impairs mitochondrial function might play a key role in the pathophysiology sepsis-AKI.

Mitochondrial quality control mechanisms, consisting of biogenesis (making new mitochondria), fission/fusion of mitochondria and mitophagy (removal of damaged mitochondria), can counteract mitochondrial damage and subsequent dysfunction [7, 8, 43]. Sepsis-AKI patients had lower mRNA expression of *TFAM* (marker for biogenesis) and *PINK* and *PARKIN (*markers for mitophagy), but no change in mRNA expression of *MFN2* and *DRP1* (markers for fusion and fission). HUVECs induced with LPS for 48 h showed a similar pattern, where *TFAM* was decreased, but no change in *PINK*, *MFN2* or *DRP1*. Similar to our findings, sepsis is associated with increased mRNA expression and protein levels of *TFAM* in muscle, suggestive of biogenesis and a lowered mitochondrial mass [11]. Adequate compensation of mitochondrial damage seems to play an important role in determining the outcome of sepsis, as mRNA expression of *Peroxisome Proliferator-activated Receptor Gamma Coactivator 1-alpha* (*PGC1α*) (marker of biogenesis upstream of *TFAM*) is associated with survival from sepsis [11]. Also, LPS-induced mice with AKI suffer from decreased mRNA expression of *PGC1α* in the renal cortex, whereas overexpression of *PGC1α* shows recovery from LPS-induced AKI [15]. The protective role of mitophagy is illustrated by *PARK2*-deficient mice, which exhibited degradation of mitochondrial functions and impaired recovery of cardiac contractility in sepsis [44]. Additionally, inhibition of mitophagy in mice increases the sensitivity of multiple organ failure and death from sepsis [44, 45]. Here, we found a decreased expression of *PINK1* and *PARKIN*, the main mitophagy regulators, in the septic kidney, which suggests that removal of damaged mitochondria by mitophagy is impaired. Lastly, we found no changes in mRNA expression *DRP1* or *MFN2* (markers for fission/fusion) in renal biopsies or LPS treated HUVECs. Accordingly, fission and fusion did not differ between septic patients and controls in human PBMCs [46]. However, since *PINK1* and *PARKIN* mediate degradation of *MFN2* and activation of *DRP1* to prevent fusion while promoting fission [47, 48], we cannot conclude that protein levels or activity of *PINK1* and *PARKIN* and hence fusion/fission are unaltered in sepsis. Together, sepsis is associated with mtDNA damage in sepsis without upregulation of genes encoding for mitochondrial quality control processes to safeguard the mitochondria.

A strength of our study is the use of fresh direct postmortem kidney biopsies from sepsis-AKI patients and control subjects, which allowed us to directly investigate the effect of sepsis on mitochondria in the kidney in association with immunohistochemical analysis, albeit in a small cohort of patients, while other studies estimate kidney and mitochondrial damage indirect using surrogate markers in urine or blood. However, we could only analyze non-survivors, which represent the most severe critically ill patients, as it would be ethically unacceptable to obtain renal biopsies from living patients with sepsis-AKI. Postmortem changes in mRNA expression should be taken into consideration when interpreting our data, but are unlikely to have been of major relevance, as samples were collected as quickly as possible after death (24–150-min postmortem), and the kidney is among the less susceptible organs to changes in RNA integrity and gene expression as demonstrated up to 24-h postmortem [49]. Lastly, there is uncertainty as to whether renal cell carcinoma might have affected mitochondrial mass or DNA levels in surrounding healthy tissue. However, mtDNA copy number, DNA content and activities of mitochondrial enzymes are shown to be reduced within RCC tissue [50, 51]. Furthermore, impairment of mitochondrial DNA levels and mass depends on RCC aggressiveness, a trend that is not found in healthy tissue within the same kidney [52, 53]. Thus, in contrast to effects of cancer on mitochondrial mass and DNA within the tumor, this does not seem to be the case for surrounding tissue. However, even if mitochondrial mass and DNA in surrounding tissue would have been affected by the tumor, this would have led to an underestimation of the effect of sepsis and thus not compromise our findings.

## Conclusion

Our findings shed new light on the contribution of mitochondria in the pathophysiology of sepsis-AKI. We reveal that sepsis induces oxidation of nuclear and mitochondrial DNA and mtDNA damage, without signs of upregulation of mitochondrial quality control mechanisms, resulting in a reduced mitochondrial mass in the septic kidney. These findings are of clinical relevance, as sepsis is the major cause of AKI and death in critically ill patients. Dissecting the molecular mechanisms leading to mitochondrial dysfunction in sepsis-AKI is crucial for the development of novel targeted therapies to prevent or treat sepsis-AKI and potentially improve the survival. Since it is not known whether mtDNA damage is repaired after survival from sepsis, our findings might also be of relevance for long-term outcomes of sepsis. Given the role of mitochondria in the pathophysiology of sepsis-AKI, pharmacologic strategies directed at maintaining mitochondrial function, limiting oxidative stress and mtDNA damage, or enhancing mitochondrial quality control to ameliorate mitochondrial damage, might successfully prevent or halt AKI in sepsis.

## Data Availability

The datasets used and/or analyzed during the current study are available from the corresponding author on reasonable request.

## Abbreviations

AKI: Acute kidney injury
APACHE: Acute Physiology and Chronic Health Evaluation
B2M: Beta 2-microglobulin
COX1: Cytochrome C oxidase I
CYTB: Cytochrome B
DRP1: Dynamin-related protein 1
HIF1α: Hypoxia-inducible factor 1-alpha
ICU: Intensive care unit
METC: Medical Ethics Review Committee
MFN2: Mitofusin 2
mnSOD: Manganese superoxide dismutase
mtDNA: Mitochondrial DNA
mRNA: Messenger RNA
ND1: NADH dehydrogenase 1
NGAL: Neutrophil gelatinase-associated lipocalin
OGG1: 8-Oxoguanine DNA glycosylase 1
PCR: Polymerase chain reaction
PGC1α: Peroxisome proliferator-activated receptor gamma coactivator 1-alpha
PINK: PTEN-induced putative kinase 1
RIFLE: Risk, Injury, Failure, Loss of kidney function, and End-stage kidney disease
ROS: Reactive oxygen species
RT-qPCR: Reverse transcription quantitative polymerase chain reaction
SAPS II: Simplified Acute Physiology Score II
SIRT1: Sirtuin 1
TFAM: Mitochondrial transcription factor A
TOM20: Translocase of outer mitochondrial membrane 20
UMCG: University Medical Center Groningen
